# Dialectics for Artificial Intelligence

**DOI:** 10.3390/e28060611

**Published:** 2026-05-29

**Authors:** Zhengmian Hu

**Affiliations:** Adobe Research, Adobe Inc., 345 Park Avenue, San Jose, CA 95110-2704, USA; zhengmianh@adobe.com

**Keywords:** Kolmogorov complexity, algorithmic information theory, concept formation, concept transmission, multi-agent alignment, compression

## Abstract

Can artificial intelligence discover, from raw experience and without human supervision, concepts that humans have discovered? One challenge is that human concepts themselves are fluid: conceptual boundaries can shift, split, and merge as inquiry progresses (e.g., Pluto is no longer considered a planet). To make progress, we need a definition of “concept” that is not merely a dictionary label, but a structure that can be revised, compared, and aligned across agents. We propose an algorithmic information viewpoint that treats a concept as an *information object* defined only through its structural relation to an agent’s total experience. The core constraint is *determination*: a set of parts forms a reversible consistency relation if any missing part is recoverable from the others (up to the standard logarithmic slack in Kolmogorov complexity). This reversibility prevents “concepts” from floating free of experience and turns concept existence into a checkable structural claim. To judge whether a decomposition is *natural*, we define *excess information*, measuring the redundancy overhead introduced by splitting experience into multiple separately described parts. On top of these definitions, we formulate *dialectics* as an optimization dynamics: as new patches of information appear (or become contested), competing concepts bid to explain them via shorter conditional descriptions, driving systematic expansion, contraction, splitting, and merging. Finally, we formalize low-cost concept transmission and multi-agent alignment using small *grounds* that allow another agent to reconstruct the same concept under a shared protocol, making communication a concrete compute-bits trade-off.

## 1. Introduction

To build artificial intelligence, it is useful to look at human intelligence, not as an ideal to imitate in every detail, but as evidence that certain mechanisms are possible. Human concepts are both reusable and revisable. From finite and partial experience, humans form concepts such as “water”, “animal”, or “price” that can be applied to new cases to explain, predict, and guide action. Yet concepts can also change as inquiry progresses: boundaries may shift, cases may be reclassified, and previously separate notions may merge. In Ancient Greek astronomy, the “morning star” and the “evening star” were for a time treated as different objects and later unified as Venus. Similar merging and boundary revision recur throughout science.

If a concept is only a label attached to examples, the label alone cannot explain why experience does not remain a list of disconnected episodes, why independent agents with overlapping experience often reconstruct similar concepts, or why a few examples, typical features, or a short description can communicate a concept with a large extension. We therefore need a notion of concept that is tied to experience, supports revision, and makes communication cost explicit.

This paper develops such a notion using algorithmic information theory. We represent an agent’s relevant experience as a finite string *I*. Concepts are also finite strings, but a string counts as a concept only through its structural relation to *I*. The basic relation is a reversible consistency constraint called a *determination*: in a tuple of components, any missing component is recoverable from the remaining components, up to the standard logarithmic slack in Kolmogorov complexity. For the central case of a split $\left(I,P_{1},P_{2}\right)$, the pair $\left(P_{1},P_{2}\right)$ losslessly represents *I*, while each side is recoverable from the whole and the other side. Thus $P_{1}$ and $P_{2}$ are not independent names. Their identities are determined relative to the experience record *I* and the complementary side.

To compare such lossless decompositions, we use *excess information*: the extra description length paid when the whole is represented by separately described parts. Low-excess determinations provide natural decompositions, because they introduce little redundancy and little additional information beyond what is already present in the whole. In this sense, concept formation is not unconstrained naming, but a search for compact reversible decompositions of experience.

On top of this static structure, we define *dialectics*: an excess information optimization process over feasible determinations. Given a current split, a new or contested patch of information *X* may be absorbed by one side or the other. When the enlarged candidate concepts explicitly encode *X* and the corresponding determination constraints hold, the excess comparison reduces, up to logarithmic slack, to comparing the conditional description lengths of *X* under the competing concepts. The patch is assigned to the side that compresses it better under the current code. Repeated local moves can revise concept boundaries and reorganize decompositions when better codes are found.

The same framework accounts for low-cost concept transmission and multi-agent alignment. If two agents have sufficiently overlapping experience and share a public reconstruction protocol, the sender need not transmit the full extension of a concept. A small *ground* can pin down the intended side of a decomposition, and the receiver can reconstruct the concept’s extension inside its own experience record by running the shared dialectical procedure. Alignment becomes a concrete communication–computation trade-off governed by the same reversible constraints and code-length criterion.

### Contributions

- This paper gives a structural definition of concepts as components of determinations, so the claim that a string is a concept of *I* depends on a reversible information-theoretic condition rather than on a label alone.
- Excess information provides a criterion for evaluating concept decompositions by measuring the redundancy cost of splitting in description length terms.
- Dialectics is formulated as excess information optimization over feasible determinations, where concepts compete to explain new or contested information by shortening conditional descriptions, giving an operational account of concept growth and boundary revision.
- This paper formalizes low-cost concept transmission and multi-agent alignment using small grounds that allow another agent to reconstruct the intended concept under overlapping experience and a shared reconstruction protocol.

Extended Kolmogorov background, detailed proofs, related work, practical algorithmic sketches, and speculative directions are provided in the Supplementary Materials.

## 2. Background on Kolmogorov Complexity

Kolmogorov complexity formalizes the idea that an object is simple when it has a short effective description. We work with finite binary strings and fix a universal prefix-free machine *U*. The prefix-free complexity K(x) is the length of the shortest program that outputs *x* on *U*. The conditional complexity K(x∣y) is the shortest description of *x* when *y* is given as side information. The joint complexity K(x,y) denotes the complexity of a fixed computable pairing of *x* and *y*. By the invariance theorem, changing *U* changes values only by an additive constant.

Throughout, $\overset{+}{=}$ and $\overset{+}{\leq }$ hide O(log(n+1)) terms, where *n* bounds the relevant complexities. We use Kraft’s inequality and its standard counting consequences, chain rules, symmetry of information, algorithmic mutual information

I(x;y)=K(x)+K(y)−K(x,y),

conditional mutual information, and data processing. Since *K* is uncomputable but upper semicomputable, exact optimality is generally unavailable. Later arguments therefore use explicit descriptions and computable upper bounds supplied by codes, probabilistic models, MDL-style criteria, or practical compressors [1,2,3,4,5,6]. A self-contained review of these background facts is given in Supplementary Material S3.

## 3. Algorithmic Parity Structures (Determinations)

Let $x_{1:n}=\left(x_{1},\ldots ,x_{n}\right)$ be finite binary strings, with n≥2. For each *i*, write $x_{-i}$ for the tuple obtained by deleting $x_{i}$.

**Definition 1**  (Algorithmic parity structure/determination)**.** *An n-tuple $x_{1:n}$ is an* algorithmic parity structure*, or* determination*, relative to an optional context z, if every component is algorithmically recoverable from the remaining components:*

(1) $$\forall i\in \left[n\right],\;K\left(x_{i}|x_{-i},z\right)\overset{+}{=}0.$$

*Each component $x_{i}$ will be called a *concept* participating in this determination.*

The name is meant to evoke parity. For bits, if $x_{n}=x_{1}⊕\ldots ⊕x_{n-1}$, then any missing bit is determined by the others. Definition 1 replaces the XOR rule by arbitrary effective reconstruction, with reconstruction cost measured by conditional Kolmogorov complexity.

The central concept split used below is a 3-way determination $\left(I,P_{1},P_{2}\right)$. It states

$$K\left(I|P_{1},P_{2}\right)\overset{+}{=}0,\;K\left(P_{1}|I,P_{2}\right)\overset{+}{=}0,\;K\left(P_{2}|I,P_{1}\right)\overset{+}{=}0.$$

**Figure 1:**
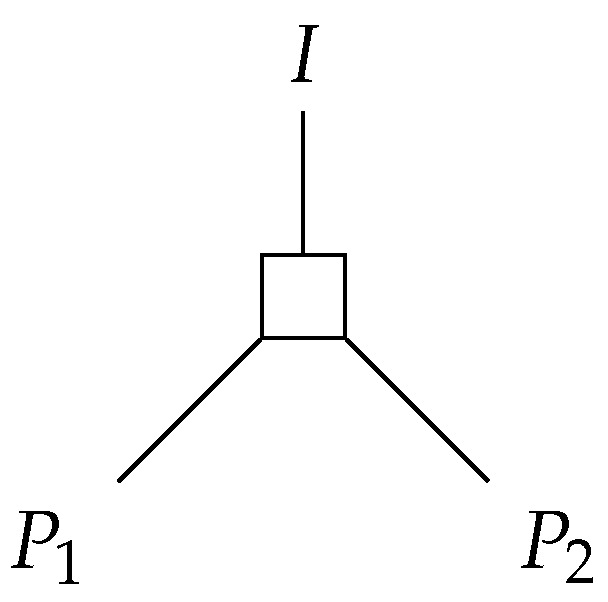
A 3-way determination $\left(I,P_{1},P_{2}\right)$. The pair $\left(P_{1},P_{2}\right)$ losslessly represents *I*, and each component is recoverable from the other two.

Thus $\left(P_{1},P_{2}\right)$ is a lossless split representation of *I*, while each side is recoverable from the whole and the other side. Figure 1 depicts this three-way determination.

In the binary-split setting used here, we call each side $P_{j}$ a concept of *I* relative to this split. The qualifier “of *I*” is essential: $P_{j}$ is not a concept by itself, but a component whose boundary and identity are fixed through a reversible relation with the whole experience *I* and the complementary side.

Finally, conditioning on a shared context *z* can be treated as ordinary participation in a larger determination: up to the same slack, an *n*-way determination relative to *z* can be viewed as an unconditional determination on $\left(x_{1},\ldots ,x_{n},z,z\right)$, since each copy of *z* is recoverable from the other.

### 3.1. One-Variable Completion

Determinations can be used as constraints. Suppose $x_{1},\ldots ,x_{n}$ are fixed and we seek one additional string $x_{n+1}$ that closes the tuple into a determination:

(2) $$\min_{x_{n+1}}\;K\left(x_{n+1}\right)\;s.t.\;\left\{\begin{array}{c} K\left(x_{n+1}|x_{1:n}\right)\overset{+}{=}0,\\ \forall i\in \left[n\right],\;K\left(x_{i}|x_{-i},x_{n+1}\right)\overset{+}{=}0. \end{array}\right.$$

These constraints are exactly the requirement that $\left(x_{1},\ldots ,x_{n},x_{n+1}\right)$ forms a determination. The feasible set is nonempty: $x_{n+1}:=\left(x_{1},\ldots ,x_{n}\right)$ is feasible. The optimal completion is a minimal piece of glue that makes all missing-coordinate reconstructions possible.

If $x_{n+1}$ is feasible, then for every *i*, $\left(x_{-i},x_{n+1}\right)$ reconstructs $x_{i}$; hence, $K\left(x_{i}|x_{-i}\right)\overset{+}{\leq }K\left(x_{n+1}\right)$, so the largest missing-coordinate conditional complexity is a necessary cost. The theorem shows that this lower bound is tight up to logarithmic slack.

**Theorem 1**  (One-variable completion cost)**.** *For any fixed n≥2 and strings $x_{1:n}$, the optimal value of* (2) *is*

$$\max_{i\in [n]}K\left(x_{i}|x_{-i}\right)$$

*up to logarithmic slack.*

The same completion problem relativizes to a fixed shared context *z*: keep the objective $K(x_{n+1})$, replace each conditional complexity in the constraints by K(·∣·,z), and require feasibility to mean that $\left(x_{1},\ldots ,x_{n},x_{n+1}\right)$ forms a determination relative to *z*. Its optimal value is

$$\max_{i\in [n]}K\left(x_{i}|x_{-i},z\right)$$

up to logarithmic slack.

This theorem shows that enforcing a reversible consistency relation has an explicit information cost. The fuller discussion of its connection to Muchnik-style multi-conditional descriptions and information distance is given in Supplementary Material S4, and the proof is collected in Supplementary Material S9 [7,8].

One-variable completion is the cleanest case: its optimal cost is the largest missing-coordinate conditional complexity. With two or more free components, completion becomes a joint optimization under determination constraints: information can be distributed across the free variables.

### 3.2. Pivoting and Concept Expansion

Determinations can be composed into networks. We next consider a basic local rewrite of such networks. Consider a two-node chain of three-way determinations sharing one bridge concept, for example (A,B,Z) and (Z,C,D). A pivot exists if there is another bridge *F* such that (A,C,F) and (B,D,F) are also determinations. Thus the same four strings A,B,C,D form one two-node chain through the original bridge *Z* and, if the pivot exists, another through the different bridge *F*. This second chain is an additional feasibility condition, not a consequence of the original chain.

**Figure 2:**
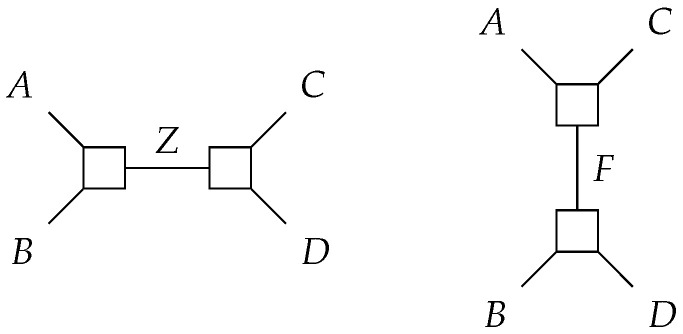
A pivot for two adjacent 3-way determinations. The left diagram is the original chain with bridge *Z*, and the right diagram is the pivoted chain with bridge *F*.

**Figure 3:**
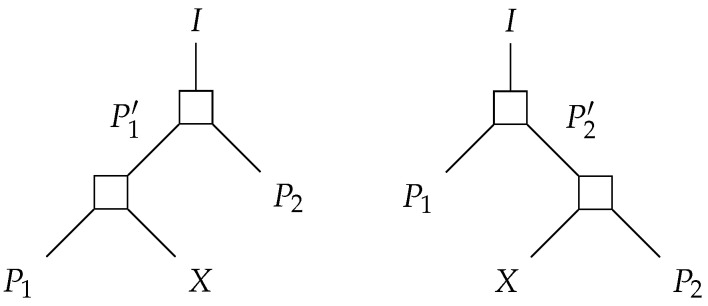
Competing absorptions of a patch. The contested patch *X* may be absorbed into $P_{1}$ or into $P_{2}$, producing two competing feasible splits.

A pivot bridge can sometimes be read as an expanded concept. In Figure 2, if *D* is interpreted as an existing concept and *B* as the information being absorbed, then the new bridge *F* in (B,D,F) can be read as *D* expanded by absorbing *B*. The competing absorptions in Figure 3 use the same two-node graph topology, with this expansion reading made explicit.

**Theorem 2**  (Pivot moves are not universal)**.** *There exist finite strings A,B,C,D,Z such that (A,B,Z) and (Z,C,D) are three-way determinations sharing the bridge concept Z, but there is no string F such that both triples (A,C,F) and (B,D,F) are determinations.*

Thus pivoting is best viewed as an opportunity rather than a universal identity. When no pivot exists, the failure indicates dependence that cannot be re-expressed through a different bridge without adding new information. Supplementary Material S4 gives the finite-field counterexample and discusses its connection to extractable common information, while Supplementary Material S9 gives the rest of the proof, including the mixing lemma.

### 3.3. Excess Information

For a three-way determination π=(A,B,C), we read (B,C) as a split representation of *A*. Since *A* is recoverable from (B,C),

$$K\left(A\right)\overset{+}{\leq }K\left(B,C\right)\overset{+}{\leq }K\left(B\right)+K\left(C\right).$$

The overhead paid by describing *B* and *C* separately is

(3) Δ(A,B,C):=K(B)+K(C)−K(A).

We call this the *excess information* of the determination. By the preceding inequality, $\Delta \left(A,B,C\right)\overset{+}{\geq }0$.

Excess information is the objective used here for evaluating lossless decompositions of a whole into parts. Since $K\left(A|B,C\right)\overset{+}{=}0$,

$$\Delta \left(A,B,C\right)\overset{+}{=}I\left(B;C\right)+K\left(B,C|A\right),$$

so the same quantity records the duplicated information incurred when *B* and *C* are encoded separately and the additional information in (B,C) beyond *A*. Thus a low-excess decomposition avoids separating parts that share substantial mutual information and avoids introducing artifacts not present in the original information *A*.

For every fixed *A* and excess budget *L*, only finitely many pairs (B,C) make (A,B,C) a determination with Δ(A,B,C)≤L. Indeed, the excess bound implies

$$K\left(B,C|A\right)\overset{+}{\leq }L,$$

so Kraft counting gives at most $2^{L+O(\log K(A))}$ such pairs. Consequently, for fixed *A* and *L*, the object of study is a finite family of feasible decompositions. For fixed *A*, this bound scales exponentially with *L*, so very low-excess budgets leave a highly constrained feasible family.

## 4. Dialectics as an Optimization Problem

We now treat concept formation and revision as excess information optimization over feasible determinations. For a fixed experience string *I*, the simplest dialectical objective is

(4) $$\begin{array}{cc} \min_{P_{1},P_{2}}\; & K\left(P_{1}\right)+K\left(P_{2}\right)-K\left(I\right)\\ s.t.\; & (I,P_{1},P_{2})\;\mathrm{is}\;a\;\mathrm{determination}. \end{array}$$

A feasible pair $\left(P_{1},P_{2}\right)$ is a lossless split representation of *I*: the whole is recoverable from the two sides, and each side is recoverable from the whole together with the other side. The objective measures the extra cost of describing the two sides as separate concepts. Since *I* is fixed, minimizing excess over feasible decompositions is equivalent to comparing $K\left(P_{1}\right)+K\left(P_{2}\right)$, the description length of representing the whole through its recoverable parts.

At the limiting ideal of unbounded computation, dovetailing over the universal description language makes the whole-object upper bound for *I* converge from above to K(I), though without a finite-time optimality certificate. At that limit, no feasible split can make $K\left(P_{1}\right)+K\left(P_{2}\right)$ fall below K(I) except up to additive slack, while $P_{1}=I$, $P_{2}=\epsilon$ already gives O(1) excess. Thus, as the attempt to understand *I* is pushed further, boundaries between concepts tend to recede: relations among the concepts are integrated into a whole-object description of *I*, so that the concepts are subsumed by that whole-object description rather than kept as separate parts.

For finite-computation implementations, the relevant quantities are achieved description lengths under concrete compressors, probabilistic models, prequential codes, or MDL-style two-part codes [5,6,9]. Under such restricted coding protocols, a nontrivial decomposition can achieve a shorter code-length than a single whole-object code, because different sides can expose different regularities and be described by methods better matched to them. Dialectics therefore compares realizable decompositions by the tracked excess information upper bounds supplied by the available coding protocol. For the local patch moves below, this finite-computation comparison is between two feasible absorptions of the same patch *X*.

### 4.1. Local Patch Competition

A common local move relocates a small information patch *X* from one concept to another. Suppose the same information object *I* admits two feasible candidate splits: $P_{1}$ absorbs *X*, producing a determination $\left(I,P_{1}^{\prime },P_{2}\right)$, or $P_{2}$ absorbs *X*, producing a determination $\left(I,P_{1},P_{2}^{\prime }\right)$. Following the pivot constructions of Supplementary Material S4, assume also that the absorption triples $\left(P_{1}^{\prime },P_{1},X\right)$ and $\left(P_{2}^{\prime },P_{2},X\right)$ satisfy the corresponding determination constraints. Holding *I* fixed, the excess objective compares

(5) $$K\left(P_{1}^{\prime }\right)+K\left(P_{2}\right)\;\mathrm{against}\;K\left(P_{1}\right)+K\left(P_{2}^{\prime }\right).$$

The difference between these two objective values can be rewritten using conditional complexities of *X*. The reduction uses the following identity for three-way determinations.

**Lemma 1**  (Complexity–difference identity)**.** *If (A,B,C) is a three-way determination, then*

$$K\left(A\right)-K\left(B\right)\overset{+}{=}K\left(C|B\right)-K\left(C|A\right).$$

Applying Lemma 1 to the two absorption triples and subtracting the resulting identities gives

(6) $$\begin{array}{cc} \; & \left[K\left(P_{1}^{\prime }\right)+K\left(P_{2}\right)\right]-\left[K\left(P_{1}\right)+K\left(P_{2}^{\prime }\right)\right]\\ \; & \;\overset{+}{=}\left[K\left(X|P_{1}\right)+K\left(X|P_{2}^{\prime }\right)\right]-\left[K\left(X|P_{1}^{\prime }\right)+K\left(X|P_{2}\right)\right]. \end{array}$$

If the enlarged candidate concepts $P_{1}^{\prime }$ and $P_{2}^{\prime }$ explicitly encode the absorbed patch *X*, then $K(X|P_{1}^{\prime })$ and $K(X|P_{2}^{\prime })$ are small. This is an additional coding assumption, not a consequence of determination alone. Under this assumption, the comparison is governed, up to logarithmic slack and the small terms $K(X|P_{1}^{\prime })$ and $K(X|P_{2}^{\prime })$, by

$$K\left(X|P_{1}\right)\;\mathrm{versus}\;K\left(X|P_{2}\right).$$

In the idealized case where the small terms are O(1), the patch is assigned to the side that gives the shorter conditional description of *X*. Operationally, concepts compete to produce shorter descriptions of the same patch under their side information, and the better-compressing side gets to incorporate it.

The local rule gives a concrete instance of the zeroth-order procedure: generate the two feasible absorptions, compare computable upper bounds on their excess information, and retain the candidate with the smaller bound. If one accepts only moves that strictly decrease the current tracked upper bound, that bound is monotone and acts as a Lyapunov quantity for the optimization dynamics. This does not make the unknown true excess information monotone, nor make assignments irreversible: later improvements in coding methods may support reassigning a patch. Since the comparisons above hold only up to logarithmic slack, a practical margin rule is to commit only when the code-length gap is large relative to expected slack and estimation noise, near ties remain revisable.

### 4.2. Grounded Dialectics

The split $\left(I,P_{1},P_{2}\right)$ is symmetric: swapping the two sides preserves both feasibility and excess information. To refer to, re-identify, or communicate one side, we introduce *grounds*: asymmetric side-information strings $\hat{A},\hat{B}$ that anchor the two sides.

For fixed *I* and grounds $\left(\hat{A},\hat{B}\right)$, define grounded dialectics by

(7) $$\begin{array}{cc} \min_{A,B}\; & K\left(A|\hat{A}\right)+K\left(B|\hat{B}\right)\\ s.t.\; & (I,A,B)\;\mathrm{is}\;a\;\mathrm{determination}. \end{array}$$

The grounds identify the two sides, and the objective encourages each side to stay close to its ground by a conditional description penalty. Any target feasible split can be pinned down trivially by taking the grounds to be the two sides themselves. The useful case is when small grounds still identify a split. Any optimal solution of grounded dialectics satisfies the following excess bound, showing that small grounds can only select low-excess determinations.

**Theorem 3**  (Ground complexity controls excess)**.** *Let $\left(A^{★},B^{★}\right)$ be optimal for* (7)*. Then*

$$K\left(\hat{A}\right)+K\left(\hat{B}\right)\overset{+}{\geq }K\left(A^{★}\right)+K\left(B^{★}\right)-K\left(I\right).$$

Grounded dialectics can be used as a communication mechanism between different agents. The setup requires sufficiently overlapping experience and a shared deterministic reconstruction protocol with fixed compute budget. The sender transmits grounds, and the receiver runs the protocol to reconstruct the intended side in its own experience record. Thus the sender need not transmit the full extension: a small ground can communicate a large concept.

### 4.3. Boundary-Mediated Splits

Many concrete segmentation-like problems include an explicit boundary object *B*: a curve, binary mask, set of polygon vertices, text span, or time-frequency mask. In this regime, *B* is the main decision variable: once *I* and *B* are fixed, $P_{1},P_{2}$ are induced as algorithmic restrictions of *I* to the two sides. The standard boundary assumptions for this regime are

$$K\left(B|P_{i}\right)\overset{+}{=}0,\;K\left(P_{i}|I,B\right)\overset{+}{=}0\;\left(i=1,2\right),\;K\left(I|P_{1},P_{2}\right)\overset{+}{=}0.$$

These assumptions say that the boundary is recoverable from either side, the parts are computable from the whole and the boundary, and the two parts cover the whole. Together they imply that $\left(I,P_{1},P_{2}\right)$ is a determination.

The split is scored by the excess information

$$\Delta :=K\left(P_{1}\right)+K\left(P_{2}\right)-K\left(I\right).$$

Under the boundary assumptions, the same objective can be written as

$$\Delta \overset{+}{=}K\left(B\right)+I\left(P_{1};P_{2}|B\right)+K\left(B|I\right).$$

This form separates three sources of overhead: boundary complexity, residual dependence between the two sides after the boundary is known, and ambiguity of the boundary given the whole. Other equivalent accounting forms, implication relations, and diagrams are deferred to Supplementary Material S5.

For a local boundary move, let *X* be a contested block near the boundary. Assume *X* lies outside both current sides. The two candidate assignments are

$$\left(P_{1}^{\prime },P_{2}\right)\;\mathrm{with}\;\mathrm{boundary}\;B_{1},\;\left(P_{1},P_{2}^{\prime }\right)\;\mathrm{with}\;\mathrm{boundary}\;B_{2}.$$

Here $P_{i}^{\prime }$ denotes $P_{i}$ enlarged by including *X*. Let

$$\Delta_{1}\overset{+}{=}K\left(P_{1}^{\prime }\right)+K\left(P_{2}\right)-K\left(I\right),\;\Delta_{2}\overset{+}{=}K\left(P_{1}\right)+K\left(P_{2}^{\prime }\right)-K\left(I\right)$$

be the corresponding excess information values. Since *I* is fixed,

$$\Delta_{1}-\Delta_{2}\overset{+}{=}\left[K\left(P_{1}^{\prime }\right)-K\left(P_{1}\right)\right]-\left[K\left(P_{2}^{\prime }\right)-K\left(P_{2}\right)\right].$$

Using the structured assumptions and derivation given in Supplementary Material S5, this becomes

(8) $$\Delta_{1}-\Delta_{2}\overset{+}{=}\left[K\left(X|P_{1}\right)-K\left(X|P_{2}\right)\right]+\left[K\left(B_{1}|P_{2}^{\prime }\right)-K\left(B_{2}|P_{1}^{\prime }\right)\right].$$

The first bracket is the content bid: it favors assigning *X* to the side that compresses it better. The second bracket is the boundary bid: it penalizes the assignment that leaves a more complex or less visible boundary. When the boundary terms are negligible or comparable, the rule reduces to assigning *X* to the side with the shorter conditional code $K(X|P_{i})$.

## 5. Positioning and Conclusions

The framework is closest in spirit to MDL and related coding approaches: good explanations are evaluated by the description lengths they achieve under an effective code [5,6]. It also relates to compression-based clustering and normalized information distance, where similarity is measured by shared description length [10,11]. The difference is that the present object is neither merely a pairwise distance nor a fixed model class, but a reversible decomposition constrained by determinations.

Under restricted computable code families, classical clustering, mixture modeling, and segmentation instantiate the same competitive-coding rule in different forms. In *k*-means, assigning a point to the nearest centroid is equivalent, up to constants, to choosing the cluster with the smallest squared-error coding surrogate [12,13]. In EM for mixture models, components compete through posterior responsibilities computed from mixture weights and likelihoods, while negative log-likelihood provides the corresponding code-length objective [14]. In boundary-based segmentation, regions compete by the joint cost of coding region content and boundary structure [15,16]. These methods do not use near-optimal Kolmogorov codes, but they instantiate the same competitive compression pattern. In this sense, dialectics does not replace these methods. It abstracts their shared structure by separating the objective, the computable code family used to upper-bound it, and the local move protocol used to compare feasible solutions.

We have described concepts as information objects tied to experience by reversible determinations. Excess information measures how expensive it is to represent a whole by separately described parts, and dialectics is the resulting excess information optimization dynamics: in local patch moves where the competing absorptions remain feasible and the enlarged concepts explicitly encode the contested patch, the comparison reduces, up to logarithmic slack, to conditional description lengths for that patch. Grounds provide an alignment mechanism: under overlapping experience and a shared reconstruction protocol, compact grounds can pin down intended concepts and support their reconstruction.

A natural next step is to scale compute for dialectics over larger experience records, so that previously unnamed concepts can be searched for as new low-excess determinations. Extended discussion is left to the Supplementary Materials.

## Data Availability

No new data were created or analyzed in this study.

## Supplementary Materials

The following supporting information can be downloaded at: https://www.mdpi.com/article/10.3390/e28060611/s1, Supplementary Material S1, guide, S2, extended motivation, S3, Kolmogorov background, S4, determinations and pivot moves, S5, dialectical optimization and grounds, S6, related work, S7, practical methods, S8, future directions, S9, proofs, S10, limits of computable code-length upper bounds, S11, notation table, and S12, references. Additional references cited in the Supplementary Materials are included here [17,18,19,20,21,22,23,24,25,26,27,28,29,30,31,32,33,34,35,36,37,38,39,40,41,42,43,44,45,46,47].
